# What's in a Name? Can Mullein Weed Beat TB Where Modern Drugs Are Failing?

**DOI:** 10.1155/2011/239237

**Published:** 2010-09-19

**Authors:** Eibhlín McCarthy, Jim M. O'Mahony

**Affiliations:** Cork Institute of Technology, Rossa Avenue, Bishopstown, Cork, Ireland

## Abstract

Common mullein weed (*Verbascum thapsus*) has a large number of synonyms and old local “nick names” which connect the plant with mycobacteria. A strong history of medicinal use has been uncovered for the treatment of tuberculosis, tubercular skin disease, leprosy, and mycobacterial disease in animals. Here, we examine problems encountered in treating such diseases today, the historical and scientific links between mullein and pathogenic bacteria, and the possibility that this common weed could harbour the answer to beating one of the world's biggest infectious killers.

## 1. Tuberculosis: Modern Day Scourge

It has killed ancient Egyptians and Iron Age British settlers as well as John Keats, D. H. Lawrence, and Vivien Leigh. Throughout history, it has left its mark on our ancestors as “phthisis”, “consumption”, and the “white plague”. But far from being a disease of the past, Tuberculosis (TB) is now killing almost 5000 per day, or one person about every 20 seconds [1]. The World Health Organisation (WHO) has declared a global emergency [2] and as antibiotic resistance increases [3], the hunt for new treatments continues.

## 2. Treating Tuberculosis—Present and Future

Mycobacteria are notoriously tough micro-organisms. Their intrinsic resistance to many antibiotics can be largely attributed to the unique structure of the cell envelope, which is rich in high molecular weight fatty acids and peptidoglycan cross-linkage [6]. Permeability is mainly restricted to lipophilic molecules [7] such as the anti-TB agent Rifampicin. This envelope also seems to be a dynamic structure—changing according to whether the bacterium is growing or persisting in host tissues [8]. A combination of antibiotics is therefore required to treat mycobacterial infection. Current Irish recommendations are that all new TB patients (including HIV positive) who have not previously been treated with anti-TB drugs receive the internationally accepted first line treatment regimen outlined by the WHO [9]. This includes initial treatment with Isoniazid, Rifampicin, Pyrazinamide, and Ethambutol for two months, followed by a four-month continuation phase of further treatment with Isoniazid and Rifampicin. In the case of a 60 kg adult, this equates to over half a kilogram of antibiotics. Monthly sputum samples are examined for the continued presence and/or reappearance of tubercle bacilli—in this case, treatment needs to be modified due to resistance and may be extended to 9 months or longer. Extensively drug-resistant TB (often incurable) first emerged in 2006 [10] and has now been reported in over 50 countries worldwide [1]. The WHO recently reported that in some areas of the world, one in four people with TB becomes ill with a form of the disease that can no longer be treated with standard drugs [11]. 

Three of the four first-line antibiotics listed above work by directly or indirectly affecting the synthesis of the mycobacterial cell wall and this remains an area of pharmaceutical interest [12]. The three compounds which are currently in phase II/III clinical development (namely moxifloxacin, PA824, and TMC207) elicit their activity by the inhibition of enzymes involved in cell division, respiration, and mycolic acid synthesis [13, 14]. While these new drugs were synthetically engineered, natural compounds are once again playing a bigger role in the fight against TB. Plant extracts have been shown to be capable of inhibition comparable to that of Streptomycin [15], have shown activity against multidrug resistant TB [16], and have been shown to improve the immune response to TB both in vitro and in clinical trials [17, 18]. Pharmaceutical science is increasingly beginning to realise that the “silver bullet” compounds we are attempting to engineer may have been present in nature all along.

## 3. Nature as a Source of Medicinal Compounds

Up to half of pharmaceutical agents prescribed today contain at least one ingredient derived from plants [19]—an indication that mainstream medicine has become more receptive to the use of drugs derived from botanical sources. Some of the more well-known medicines entrusted to us by Mother Nature include the analgesics codeine, salicin and morphine, the antimalarial artemisinin, the antihistamine ephedrine, and the cardiac drug digoxin. In many cases, history has given scientists clues as to which plants may contain therapeutic compounds. Of the 119 plant-derived pharmaceutical medicines recently examined by the WHO, about 74 percent are used in modern medicine in ways that correlate directly with their traditional uses as herbal medicines by native cultures [20]. As a result, scientists involved in the hunt for new anti-TB drugs are beginning to look to the past for inspiration. The Global Alliance for TB Drug Development (TB Alliance) recently announced a partnership with the Institute of Microbiology of the Chinese Academy of Sciences (IMCASs) to discover and develop promising, novel, and antituberculosis agents from natural sources [21]. The Chinese are by no means the only nationality to use traditional herbal remedies, nor the only nationality to record them. The scientific discipline known as ethnopharmacology aims to use the knowledge assembled by indigenous peoples worldwide regarding the plants they have used as traditional remedies in their own areas [22, 23]. Ireland is a country with a well-documented history of tubercular disease and leprosy [24]. To date, however, no research has been carried out into Irish traditional remedies for mycobacterial diseases such as TB.

 Ireland has a relatively low number of native vascular plant species, at 815 [25]. During a review of those plants most used in traditional Irish medicine, one repeatedly showed links to TB and other diseases caused by mycobacteria. This plant is *Verbascum thapsus*, or common mullein. 

## 4. Irish Mullein Weed—Notes from History

**Figure 1:**
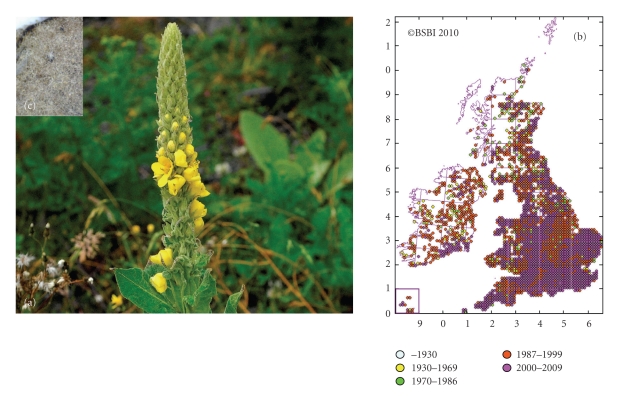
Appearance and distribution of Great Mullein (*Verbascum thapsus*). (a) *Verbascum thapsus* (Great Mullein). Photograph by Zoë Devlin [4]. (b) For the purpose of botanical distribution records, the British Isles is divided into hectads (10 km^2^ areas). Each dot on the map indicates that mullein has been identified in that hectad. As botanical record-keeping is ongoing, hectad distribution maps change over time [5]. (c) Detailed photograph of dried Mullein leaf. The branched hairs which cover the leaves and help the plant to retain water can clearly be seen. Photograph by Dr. Jim O'Mahony.

Mullein has grown wild throughout Ireland for centuries—on walls, wasteland, quarries, and roadsides. It can grow to heights of up to two metres, with a round woolly stem, broad basal leaves, and yellow flowers which bloom from June to September (Figure 1(a)) [4, 26]. This physical description explains many of the synonyms used for this plant. These include Candlewick Plant, Torches, Our Lady's Flannel, Shepherd's Staff, and Beggar's Stalk [27]. Census catalogues of the Irish flora, vice-county maps, and hectad maps all show the widespread distribution of mullein in Ireland [5, 28] (Figure 1(b)). Upon reviewing Irish traditional medicine, mullein soon becomes apparent as a leading remedy in the treatment of tuberculosis. Even up until the relatively recent introduction of antimycobacterial drugs, mullein was cultivated on a large scale in this country and even sold in the capital's best chemist shops [29]. A simple “pharmaceutical trial” in the late nineteenth century showed that the herb was beneficial in cases of tuberculosis. Dr. Quinlan of St. Vincent's hospital (Dublin, Ireland) noted mullein as “a trusted popular remedy in Ireland” and reported positive findings in 6 out of 7 cases where the herb was used to treat tubercular patients [30]. 

 The first English language herbals—written as far back as the sixteenth century—also point to mullein as a TB remedy and praise it's soothing and expectorant properties. Culpeper advises that mullein will help a persistent cough, tumours, or inflammations of the throat [31], while Gerard noted antibacterial properties of the plant, commenting on its use as a food preservative [32]. In more recent times, the extensive herbal collection by Grieve names mullein as a “remedy of the greatest antiquity for coughs....cultivated in gardens for the purpose.... in steady demand by sufferers from pulmonary consumption” and notes that it “controls the hacking cough of consumption....and bleeding of the lungs” [27]. The fine hairs which cover the surface of the mullein leaf (Figure 1(c)) may themselves have encouraged expectoration from the lungs, however, most traditional remedies encourage straining the leaf decoction to remove these before drinking [27]. The herb's potential was also noted across the Atlantic. King's American Dispensatory records that it is useful in cases of “protracted cough and haemoptysis” [33] while the herbalist Edward Shook went so far as to say that TB could be cured in its early stages using this herb alone [34].

While its historical ties with tuberculosis abound, perhaps even more intriguing is the mullein weed's apparent association with the genus *Mycobacterium* in general. While revising historical remedies, we must consider our modern day understanding of microbiology. It is now possible to prove that diseases which were once believed to be completely unrelated are in fact caused by the same pathogen. Mycobacteria are an example of this—causing a variety of infections in humans and animals (Table 1).

**Figure 2:**
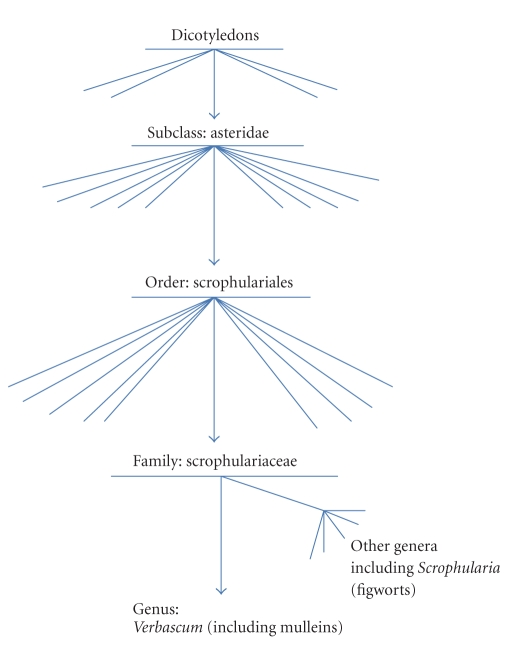
Taxonomy of the Scrophulariaceae. Flowering plants are divided into Monocotyledons and Dicotyledons on the basis of differences in their seeds. The Asteridae, a subclass of the Dicotyledons, contain a large number of medicinal plants. Of particular interest here is the family Scrophulariaceae, to which the mulleins (*Verbascum spp*) belong. (Lines represent other members at upper levels. Names are omitted for clarity).

Mullein is a member of the Scrophulariaceae. This botanical family took its name from *Scrophularia*, or figwort—a typical member of the family which was used to treat the tuberculous infection of the lymph nodes of the neck known as scrofula [35]. This name was in turn derived from the Latin scrofa, meaning “breeding sow”. The connection may be because the glands associated with the disease resemble the body of a sow or because pigs were thought to be prone to the disease. The use of the botanical name *Scrophularia* can be traced back at least five hundred years—it was used by the German botanist Otto Brunfels in 1532 [36], but the name may have been in use for some time previously. The 18th century botanists Tournefort (1719) and Linnaeus (1753) went on to clearly define the genus, giving figwort the official binominal title *Scrophularia nodosa* [37]. At that time, belief in the Doctrine of Signatures was still widespread. This was the concept that God himself had “signed” plants which could cure diseases with features of the disease itself or the organ it affected. In this case, figwort and related plants were reputed to cure scrofula because their nodular roots resembled the diseased lymph nodes [38]. Taking physical characteristics and medicinal uses into account, Linnaeus first proposed the family Scrophulariaceae (Figure 2). 

In a study of the common names of British plants, Prior states that the word Mullein translated to Moleyn in Anglo-Saxon and Malen in Old French, derived from the Latin malandrium—as was the term “malanders”, an old term for leprosy. He also notes that “the term “malandre” became also applied to diseases of cattle, to lung diseases among the rest, and the plant being used as a remedy, acquired its name of “Mullein” and “Bullock's Lungwort”” [39]. This links the mullein weed to leprosy and bovine TB. These conditions—along with scrofula—are now known to be caused by Mycobacteria. The early herbals also support a connection with bovine TB—John Parkinson gave broth of mullein to cattle suffering from coughs in the seventeenth century [40]. Finally, in Cameron's Gaelic names of plants, the synonym cow's lungwort is recorded while a Gaelic name for mullein is given as Cuingeal—possibly derived from cuing, meaning “shortness of breath” [41].

## 5. Investigating Mullein

The extensive historical use of mullein has inspired scientists to investigate the plant further. To date, a number of the ancient claims have actually proved to be true.

Modern day European complimentary medicine frequently hails mullein flower oil as a remedy for earache [42]. Trials have shown a statistically significant improvement in ear pain associated with acute Otitis Media when treated with the infusion [43]. The proven anti-inflammatory action of the constituent verbascoside [44] is the likely cause for this successful treatment though antimicrobial agents may also play a part. Extracts of the mullein leaf have also been shown in laboratory studies to possess antitumour, antiviral, antifungal [45–48], and—most interestingly for the purpose of this paper—antibacterial properties. Turker and Camper showed aqueous Mullein leaf extracts to be effective against gram positive and gram negative microorganisms, with the activity against *Klebsiella pneumoniae* rivalling that of the Erythromycin control [49]. To date, activity against Mycobacterium species has not been determined. 

## 6. Battles in Nature: Vascular Plants versus Mycobacteria

In the absence of an acquired immune system such as that possessed by humans, plants instead rely on an enormous variety of small-molecule antimicrobials. Over 100,000 such compounds are synthesised by plants [57] and activity is particularly evident against gram positive bacteria [58]. This is not surprising, as the most common bacteria in soil come under the genera Bacillus, Clostridium, Corynebacterium, and Mycobacterium—all of which are gram positive rods. Projects in natural product discovery are increasing following a recent dip [59] and many compounds with antimycobacterial properties have been uncovered from natural sources [60, 61]. In 2000, Newton reported that on a review of literature available at that time, extracts from 138 plant species and 112 pure metabolites had moderate to high antimycobacterial activities [62]. In the intervening years, ethnopharmacological studies have continued to add to the pool of natural antimycobacterials, with promising compounds extracted from the traditional remedies of countries as far removed as Mexico [23], Ethiopia [63], and India [64]. A 2004 review [60] divided the most promising botanical compounds tested by in vitro bioassays against *Mycobacterium tuberculosis* as follows.

1. Alkaloids.
2. Flavones, coumarins, chromone, and chalcone.
3. Terpenoids.
4. Steroids and saponins.
5. Phenols and polyphenols.

Compounds from each of the above classes have also been isolated from *Verbascum* species—which includes the “mulleins”.

## 7. The Pharmacological Potential of Mullein

As already outlined, the mulleins (*Verbascum spp*.) have long been known to possess expectorant and demulcent properties—these are now attributed mainly to its mucilagenous constituents and saponins [65]. Although to date mullein has not been studied in depth against mycobacteria, antibacterial efficacy against other bacteria has been proven [49] while extracts of other *Verbascum* species endemic to Turkey have activity against the *Mycobacterium smegmatis* model [66]. In particular phytochemical investigations have uncovered a diverse range of compounds within *Verbascum* species—both from whole plant [50–53] and specific parts [54–56] (Table 2). Lipophilic compounds are particularly abundant. 

As a family, the Scrophulariaceae (to which *Verbascum* species belong) harbour the chemotaxonomic markers of verbascoside, aucubin, and catalpol [67]. The latter are iridoids—a class of secondary metabolites found in a wide variety of medicinal plants, most often bound to glucose. Hydrolysis of the glycosidic bond is thought to be a prerequisite for biological activity [68]. Plants produce iridoids as a defence against infection by micro-organisms; antimicrobial action is amongst their wide range of bioactivities. The compounds shown to be common amongst *Verbascum* species are examples of this. The hydrolysis products of aucubin and catalpol, and aucubin itself, have been shown to have antimicrobial activity [69, 70]. Verbascoside has been investigated further. It has been shown to be effective against a wide range of bacteria [71] but is particularly active against gram positive bacteria such as *Staphylococcus aureus*, including drug resistant strains [72]. One possible mode of action is the inhibition of leucine uptake and hence protein synthesis [73]. Furthermore, Funes et al. have recently shown that verbascoside can disrupt the phospholipid/water interface of bacterial membranes [74].

Saponins are also a major chemical constituent of mullein. This diverse class of compounds are derived from a 30-carbon precursor and perform a range of biological actions. While previous studies have shown weak antimicrobial activity and often only at low cell density [46, 75], some of the most promising activities have been against mycobacteria [76].

## 8. Future Potential

It would appear that a member of the Scrophulariaceae which was once known as “bullock's lungwort” and has now been proven to be rich in lipophilic molecules is an ideal place to mine for potential anti-TB agents. However, while *Verbascum* species have been shown to contain a diverse range of compounds, and similar compounds have been shown to have antimycobacterial properties, the two have yet to come together in a comprehensive study. Thorough *in-vitro* screening of the bioactivity of mullein extracts against mycobacteria is required, such as that carried out by Turker and Camper against other respiratory pathogens. When dealing with the spectrum of animal- and human-related mycobacterial diseases, continued laboratory screening of herbal extracts should be encouraged. 

It is clear that many of mullein's historical uses, synonyms, anecdotes, and local “nick names” connect this plant with mycobacteria. Of course, the historical links between mycobacteria and mullein may be coincidental. A large number of medicinal uses have been recorded for this plant while its synonyms are almost as numerous. While this is common for a traditional medicinal plant, numerous connections to one pathogen—recorded throughout history and across the world—are not. Whether the link is coincidental or rooted in true antimycobacterial actions, it merits investigation. An ancient plant, an ancient infection, an ancient link, and a modern-day cure?

## Figures and Tables

**Figure 3:**
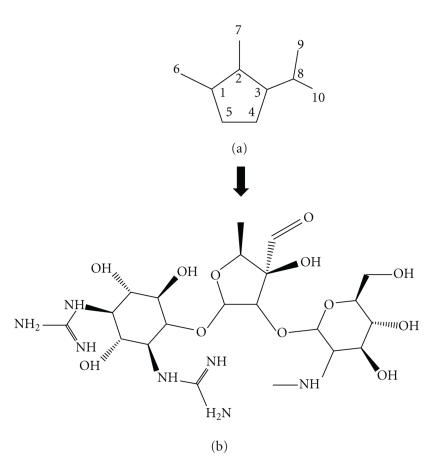
Plant iridoids and antibacterial activity. Iridoids consist of a cyclopentane ring fused to a six-membered oxygen heterocycle—Figure 3(a). Within plants, they are most often bound to glucose as glycosides. The injectable drug Streptomycin (Figure 3(b)) which can be used in the treatment of tuberculosis is glycosidal in nature. (Figure adapted from IUPAC. McNaught AD, Wilkinson A. Compendium of Chemical Terminology. Oxford: Blackwell Scientific Publications 1997).

**Table 1:** Diseases caused by Mycobacteria. Adapted from Palomino [6].

Name	Clinical form
Phthisis/Consumption/White plague	Former names for Tuberculosis (TB). Symptoms include persistent coughing, weight loss, night sweats, shortness of breath, and haemoptysis (blood in sputum)
Scrofula	TB of the neck lymph nodes, known as cervical lymphadenitis
Pott's disease	TB of the spine
Hansen's disease	Leprosy, skin lesions
Bovine TB	Tuberculosis in cattle

**Table 2:** *Verbascum* compounds. A variety of compounds have been isolated from *Verbascum thapsus* (“common mullein/great mullein”) and other members of *Verbascum* species.

Compound(s)	Verbascum species	Isolated from	Ref
Triterpenoid saponins, iridoid glycosides, steroids, sesquiterpenes, sterones	*V.thapsus*	Whole plant	[50]

Phenylethanoid glycosides, lignans, laterioside and harpagoside	*V.thapsus*	Whole plant	[51]

Siakogenins, oligosaccharides, flavones	*V.thapsus*	Whole plant	[52]

Ajugol, picroside IV, buddlindeterpene A, B and C	*V.thapsus*	Whole plant	[53]

Flavonoids and phenylethanoids.	*V.densiflorum, V.phlomoides*	Flowers	[54]

Phenylethanoid glycosides	*V.wiedemannianum*	Roots	[55]

Verbaspinoside, ajugol, phenylpropanoid glycosides	*V.spinosum*	Aerial parts	[56]
